# Atrial Fibrillation and Cancer

**DOI:** 10.3389/fcvm.2021.590768

**Published:** 2021-07-15

**Authors:** Ludhmila Abrahao Hajjar, Silvia Moulin Ribeiro Fonseca, Theuran Inahja Vicente Machado

**Affiliations:** Cancer Institute, University of São Paulo, São Paulo, Brazil

**Keywords:** atrial fibrillation, cancer, anticoagulation, cardiotoxicity, drug-drug interaction

## Abstract

Cancer patients have a higher risk of atrial fibrillation (AF) than general population, the pathophysiology mechanisms involves the pro inflammatory status of immune system in these patients and the exacerbated inflammatory response to cancer treatment and surgeries. Adequate management and prophylaxis for its occurrence are important and reduce morbidity and mortality in this population. There is a challenge in AF related to cancer to predict thromboembolic and bleeding risk in these patients, once standard stroke and hemorrhagic prediction scores are not validated for them. It is used CHA2DS2-VASc and HAS-BLED scores, the same as used in general population. In this review, we demonstrate correlated mechanisms to occurrence AF in cancer patients as well as therapeutic challenges in this population.

## Introduction

Atrial fibrillation (AF) in cancer patients is closely correlated with multiple predisposing factors and presents several mechanisms for its occurrence. The incidence of AF in cancer patients is high, with a prevalence of around 20% (1).

The mechanisms involved in the pathophysiology of these patients are related to the pro-inflammatory status of the immune system and also related to treatment, as an inflammatory response to cancer surgery, and the cardiotoxic effects of anti-cancer drugs and radiotherapy (2).

It is important to understand the mechanisms that trigger and maintain this arrhythmia in cancer patients to establish preventive measures and targeted and effective treatments.

The adequate management and prophylaxis of atrial fibrillation reduces hospitalization, morbidity and mortality of these patients. And it should be performed individually, due to the particularities that the cancer patient presents.

## Epidemiology

Epidemiological evidence is usually limited. However, cancer patients have a higher risk of AF than the general population (3).

Atrial fibrillation in the general population occurs around 1.5–2% (5).

In the cancer population, AF has an incidence of 30%. The prevalence of AF in the cancer population may vary depending on the type of neoplasm, chemotherapy treatment instituted, and surgical procedure.

The mechanisms by which certain cancer treatments trigger arrhythmia are unclear, but individual risks vary depending on the treatment, the patient's clinical circumstances, and tumor-induced metabolic and inflammatory changes.

The risk of AF is higher in patients older than 65 years and may occur in 2 out of 3 patients with cancer and those with pre-existing cardiovascular diseases (4).

Postoperative AF is the most frequent form of cancer-related AF. Its prevalence ranges from 16 to 46% for cardiothoracic surgery and 0.4–12% in non-cardiothoracic surgery (2, 8). AF occurs in about 5.6–28% of cases of lung resection in lung cancer, according to a systematic review (5). Thus, it may have a negative impact on the prognosis of these patients and may increase postoperative mortality, hospitalization time, and hospital costs (5).

Concerning to anticancer drugs, the incidence of AF secondary to treatment is between 2.2 and 16.7%. Most cytotoxic agents including alkylating agents (Cisplatin, Cyclophosphamide, Ifosfamide, Melphalan), anthracyclines, tyrosine kinase inhibitors (Ibrutinib, Sorafenib, Sunitinib), antimetabolites, taxanes, and topoisomerase II inhibitors have been found to largely induce AF cardiotoxicity (2, 6) (Table 1).

**Table 1:** Anti-cancer drugs related to atrial fibrillation.

**Alkylating agents:**
- Nitrogen mustards: Melphalan, Cyclophosphamide - Platinum complexes: Cisplatin.
**Antimetabolites:** Capecitabine, 5-Fluorouracil, Gemcitabine.
**Anthracycline agents:** Doxorubicin.
**Bruton tyrosine kinase:** Ibrutinib
**Taxanes:** Docetaxel, Paclitaxel
**HER2 inhibitors:** Trastuzumab
**Monoclonal antibodies:** Alemtuzumab, Cetuximab, Ipilimumab, Obinutuzumab, Ofatumumab, Rituximab.
**Small molecules:** Sorafenib, Sunitinib.
**Vascular endothelial growth factor inhibitors:** Bevacizumab
**Histone deacetylase inhibitors:** Dacinostat, Belinostat, Romidepsin
**Proteasome inhibitors:** Carfilzomib, Bortezomib
**Immunotherapy:** Interleukin 2
**Hormones:**
- Gonadotropin-releasing hormone (GnRH) antagonist: Degarelix - Androgen Synthesis Inhibitors: Abiraterone - Aromatase inhibitors - Glucocorticoids: high doses of Dexamethasone.

Hemorrhagic and thromboembolic events occur twice as often in cancer patients, compared to the general population when associated with the use of DOACs (1).

## Pathophysiology

**Figure 1:**
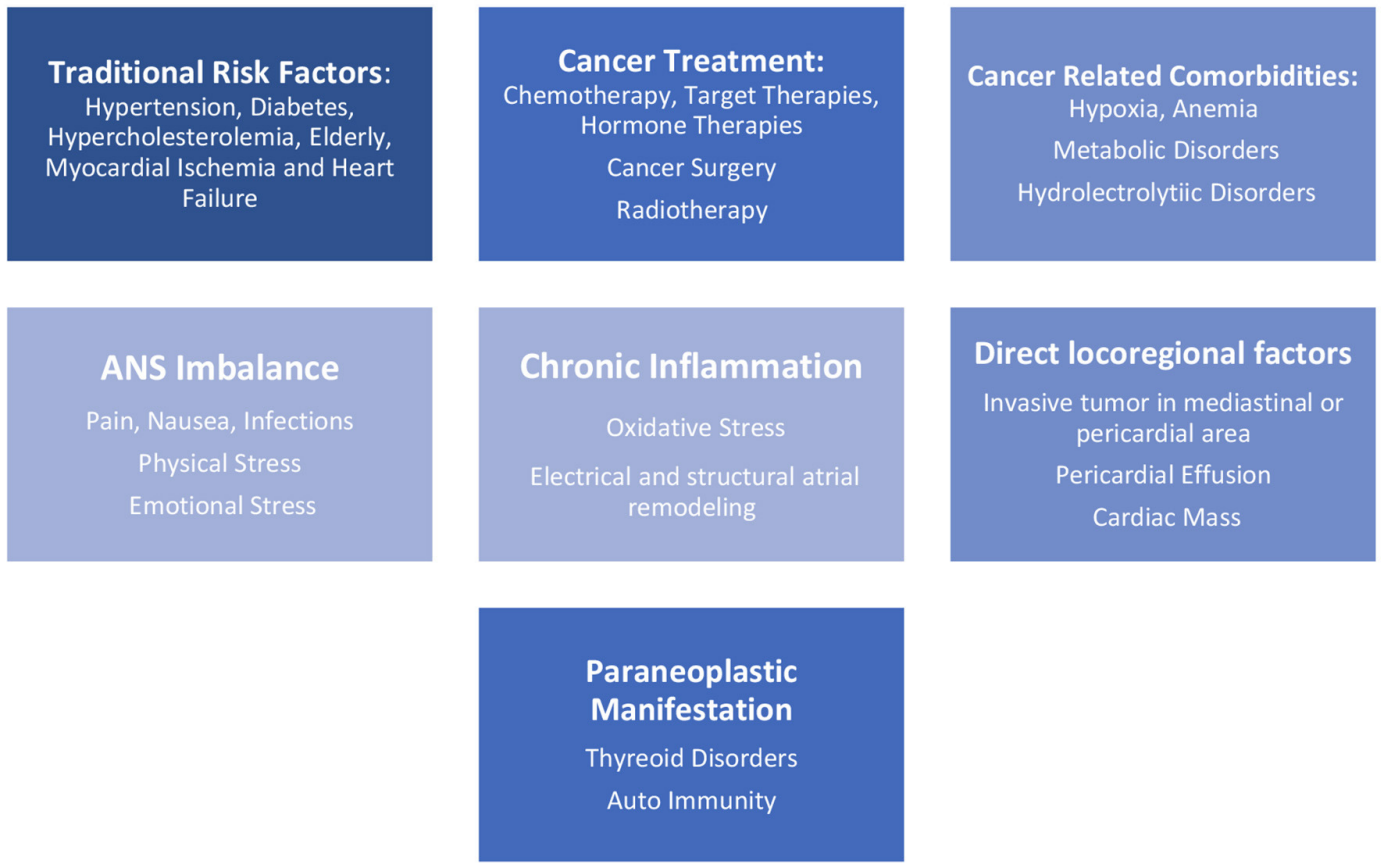
Pathophysiology of AF in cancer patients.

AF in cancer patients encompasses several risk factors, such as traditional risk factors present in the general population as hypertension, diabetes mellitus (5, 7), hypercholesterolemia, smoking status, alcohol consumption (3), heart failure, myocardial ischemia, chronic pulmonary disease, thyroid dysfunction, chronic kidney disease, and advanced age, and also inherent factors related to cancer, as hydro electrolyte abnormalities, hypoxia, and metabolic disorders (5, 7). There are other risk factors related to cancer, such as autonomic nervous system (ANS) imbalance with an increase of sympathetic stimulus caused by pain and others forms of physical or emotional stress. Cancer surgical treatments, chemo- and radiation therapies, and even the malignancies and their progression cause extreme inflammatory stress (5) and facilitate the occurrence of AF (Figure 1).

Cardiotoxicity is one of the most significant adverse effects in cancer treatment, and it is responsible for a considerable increase in morbidity and mortality. Also, the inflammatory stress caused by cancer and its treatment increases risk to unstable previous cardiovascular disease. Several modalities of cancer treatment, such as chemotherapies, radiotherapies, hormone therapies and target therapies are related to cardiotoxicity (2, 4, 10) (Table 1), and its association enhance risk of AF.

It is believed that the higher prevalence of AF in cancer patients is due to malignancy resulting in systemic inflammation that facilitates the occurrence of AF due to atrial re-structuring. To support this idea is the increased levels of C-Reactive Protein (CRP), such as Tumor Necrosis Factor α (TNF α) and Interleukins 2, 6, and 8, inflammatory markers, that are found in cancer patients and are associated with risk of this arrhythmia.

The involvement of the immune system has also been hypothesized as autoimmune paraneoplastic syndrome sustained by antibodies direct against tumor antigens and may lead to immune reaction against atrial structures that may trigger atrial fibrillation.

## Treatment

Currently, there are no specific guidelines for the management of AF in cancer patients. The treatment follows the same principles and objectives as the general population, as to improve symptoms, control arrhythmia, and prevent stroke and systemic embolism as recommended by the current guidelines in ESC and ACC/AHA (4, 5).

### Anticoagulation Therapy

The cancer patient has some particularities, such as hypercoagulable state and pro-thrombotic effect increased by some anticancer therapies, and also has an increased risk of bleeding. They are not explained by the validated thromboembolic risk assessment score (5, 6): CHA_2_DS_2_ – VASc [Congestive heart failure or left ventricular dysfunction, **H**ypertension, **A**ge 75 (doubled), **D**iabetes, **S**troke [doubled], **V**ascular disease, **A**ge 65–74, **S**ex - female]. As well as in the bleeding risk score: HAS-BLED (Hypertension, **A**bnormal renal/liver function, **S**troke, **B**leeding history or predisposition, **L**abile international normalized ratio, **E**lderly - age >65 years, **D**rugs/alcohol concomitantly) (Table 2).

**Table 2:** Thromboembolic and bleeding risk assessment score.

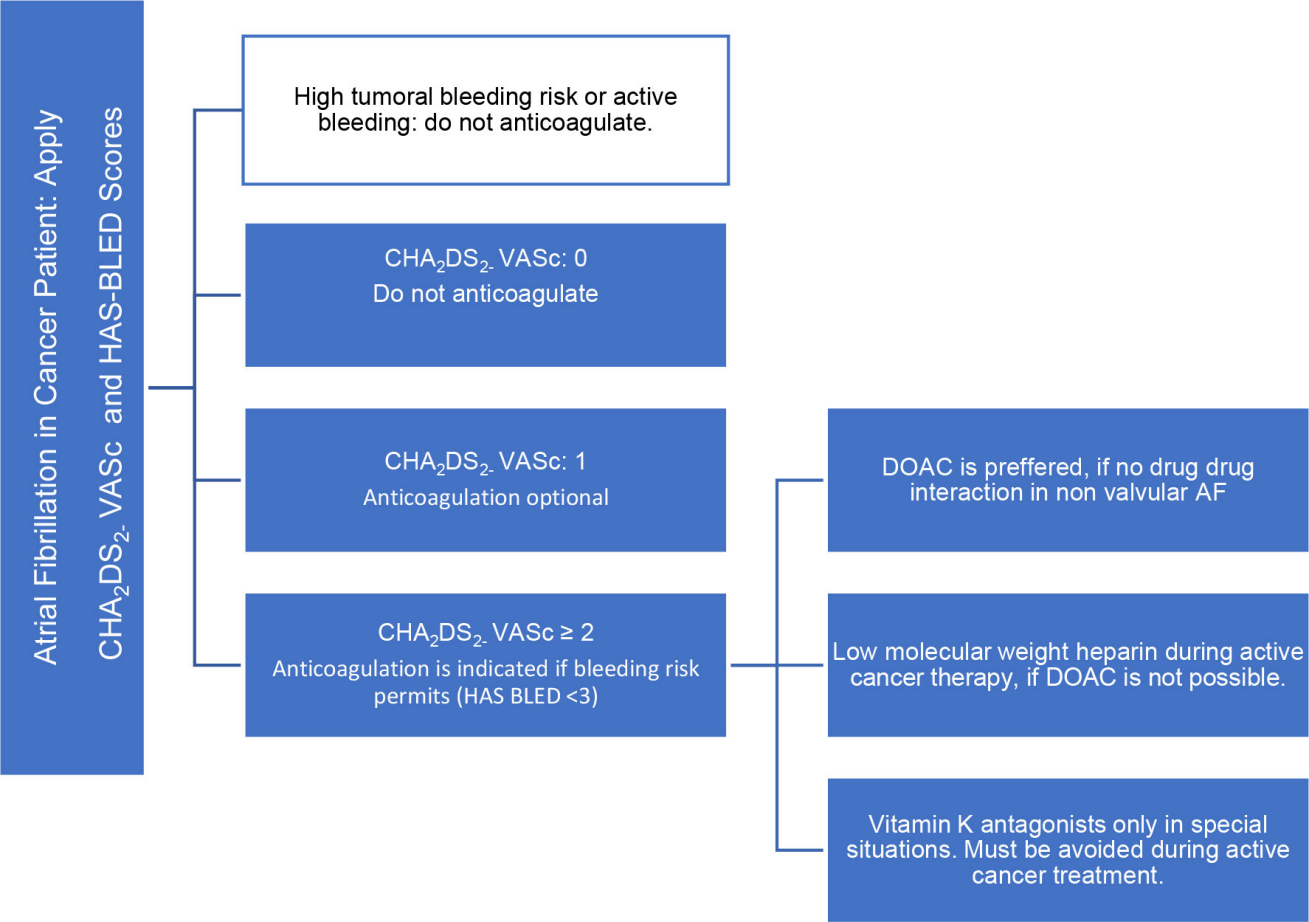

Thus, having a limited value of these scores, the analysis of patients with cancer and risk of developing AF, the decision on whether to start anticoagulation should be individualized, weighing the risks vs. benefits. It is necessary to analyze patient goals and preferences in treatment, potential drug-drug interactions, status performance, and prognosis of cancer.

The choice of anticoagulants in the treatment of AF in cancer patients is an important part of cardio-oncology field. Most of the general cardiologists treat anticoagulation similar to patients without cancer. However, it is known that in cancer patients, vitamin K antagonists (VKA) have several limitations, especially during chemotherapy, with periods of nausea and vomiting and poor food intake, as well drug-drug interactions. Currently, the percent time in the therapeutic range is poor. Moreover, their delayed onset and prolonged duration of action make the management of invasive procedures or episodes of thrombocytopenia more challenging (8).

Direct oral anticoagulants (DOACs) have the advantage of fast onset of action, short half-life, and fewer food/drug interactions than VKA. It is not necessary routine blood tests to ensure the patient is within the therapeutic window of anticoagulation. However, all DOACs are influenced by the P-glycoprotein (P-gp) system and are also subject to vary the metabolism via cytochrome P450 system (via CYP3A4) in the liver, mostly Apixaban and Rivaroxaban. The use of DOACs and drugs that are inhibitors or inducers of P-gp/CYP3A4 leads to the risk of anticoagulation levels outside of the therapeutic range. Coadministration of any DOACs is not recommended with cancer drugs and adjunctive therapies that have strong P-gp inducers or inhibitors. This is a serious limitation to their use in cancer patients, given that many chemotherapy agents fall in this category (Table 3).

**Table 3:** CYP3A4 and P-gp interaction with cancer drugs.

**Drug – drug interaction between Cancer Drugs With DOACs**	
**CYP3A4 interactions (Rivaroxaban and Apixaban)**	**P-gp interactions (All DOACs)**
• Antimitotic agents: Paclitaxel, Vinblastine	• Antimitotic agents: Vinblastine
• Anthracycline: Doxorubicin	• Anthracycline: Doxorubicin
• Tyrosine kinase inhibitors: Imatinib, Crizotinib, Vemurafenib, Vandetanib, Sunitinib	• Tyrosine kinase inhibitors: Imatinib, Crizotinib, Vandetanib, Sunitinib
• Hormone agents: Abiraterone and Enzalutamide	• Hormone agents: Abiraterone and Enzalutamide
• Immune modulating agent: Dexamethasone	• Immune modulating agent: Dexamethasone

If DOAC is not permitted, low molecular weight heparin (LMWH) is preferable over vitamin K antagonists during active cancer treatment, with more favorable results concerning interactions and therapeutic anticoagulation. The disadvantage of this medication is cost, discomfort in the application of the medication and prolonged use due to active cancer.

Basically, vitamin K antagonists are reserved in valvular AF, during non-active cancer treatment, period that has less drug-drug interaction and oral intolerance, and for renal impairment <15 ml/min.

Decisions on the choice of anticoagulant should be taken on case by case.

The ablation therapy in patients with AF and cancer is not well-defined (4).

Percutaneous left atrial appendage closure is a safe and effective procedure indicated as an alternative to anticoagulation in patients with a high embolic risk that presents contraindication for long-term anticoagulation. In cancer patients, this procedure should be considered as an option in AF not related to mechanical heart valves or to moderate to severe rheumatic mitral stenosis, with a life expectancy more than 1 year and is not permitted long-term anticoagulation (4).

### Special Situations in Anticoagulation Therapy

#### Chronic Renal Failure

DOACs are safe and effective in patients with active cancer treatment and creatinine clearance >30 ml/min. They can be used until creatinine clearance 15 ml/min, carefully monitoring, because dehydration, sepsis, and cancer drug nephrotoxicity can deteriorate renal function. Renal dose adjustment must be done (4, 9).

#### Thrombocytopenia

Is a common event during cancer therapy, and it can be developed by myeloablative chemotherapy, tumor invasion of the bone marrow, or secondary from an immune mediated phenomenon. There is no contraindication to anticoagulant therapy (if indicated) in patients with platelet counts over 50.000 μL. DOACs, LMWHs and VKA therapy are possible, reserving the particularity of each therapy, already described above. Patients with platelet account between 25 and 50.000 μL in patients with high thrombotic risk (mechanical heart valves, rheumatic mitral stenosis, previous systemic embolism) can be treated with a low dose of LMWHs. Platelet count <25.000 μL must have individualized treatment (4).

#### Antiplatelet Therapy

This combination therapy during cancer treatment is indicated when the association of AF with the acute coronary syndrome (ACS) or elective percutaneous coronary intervention (PCI). Should be considered the ischemic risk and bleeding risk in each patient, particularly with gastrointestinal and genitourinary or central nervous system cancer (4). Triple therapy, AAS + Clopidogrel + Oral Anticoagulation (OAC), for at least 1 month in patients with ACS and can be extended up to 3–6 months for patients with high ischemic risk and low bleeding risk (4). It is reserved 1 month only of triple therapy in elective PCI if ischemic risk > bleeding risk. If the bleeding risk is higher, only double therapy since the PCI, with Clopidogrel and OAC. Double therapy, Clopidogrel and OAC, should maintain combination until 12 months are completed. Oral anticoagulation alone can be continued after 1 year of ACS or elective PCI in AF cancer patient (4). It is important to know that Clopidogrel is preferred over others P2Y12 in combination therapies because it has a lower bleeding risk. If the only oral anticoagulation possible is VKA therapy, rigorous monitoring of INR values (2-2,5) is needed. DOACs is preferred. Some recent trials in the general population, PIONEER AF-PCI (11), REDUAL PCI (12) and AUGUSTUS (13) trials, support the safety of Rivaroxaban, Dabigatran, and Apixaban as respective alternatives for dual therapy with Clopidogrel after percutaneous coronary intervention (PCI).

#### Chronic Liver Dysfunction

Patients with CLD were excluded from randomized clinical DOAC trials, leading to a lack of safety data in this population. Current recommendations for the use of DOAC therapy are based on data in pharmacokinetic studies and small observational studies. Rivaroxaban and Edoxaban can be prescribed with caution in patients with mild liver impairment and must be avoided in moderate or severe liver impairment. Apixaban and Dabigatran can be used with caution in mild and moderate liver impairment and must be avoided in severe impairment (14, 15). Close monitoring for signs and symptoms of bleeding is needed in these patients. Further studies are needed.

### Antiarrhythmic Therapy

The decision about antiarrhythmic therapy is part of AF treatment. Initially, treat AF triggers, as hydro electrolytic disturbance, fever, sepsis, pain and hypoxemia (4), during cancer therapy is important because sinus reversion can occur spontaneously. In an echocardiogram, it is possible to assess other potential triggers such as acute ventricular disfunction, pulmonary thromboembolism, pericardial effusion, and cardiac tamponade, tumor invasion e endocarditis. If AF persists, the decision of rate control or rhythm control must be based in check potential interactions between antiarrhythmics and cancer drugs, and also contraindications to long-term anticoagulation therapy. Ablation therapy in patients with AF and cancer is not well-defined.

## Conclusion

Atrial fibrillation has a higher incidence in cancer patients. Cancer medical treatment and surgery contribute to its occurrence, although an increased incidence of AF is observed in these patients even in the absence of treatment. This suggests that the pro-inflammatory status in cancer predispose the arrhythmia.

Common risk stratification scores, as CHA_2_DS_2_VASc and HASBLED, are not validated to this population, once do not take cancer as a variable account. An individualized stratification tool for this specific population to have a better evaluation of thrombotic and bleeding risk in cancer patients is necessary.

The anticoagulation decision is also a challenge due to drug-drug interactions and special situations as thrombocytopenia. It is a challenge to manage stroke prevention in patients with AF and cancer with antithrombotic therapies due to a lack of evidence and guidelines to guide the ideal treatment, given the complexity of these patients.

AF brings an increase in the morbidity and mortality of these patients, in addition to affecting the prognosis, the therapeutic effects, increasing the costs of hospitalization and disability of these patients.

Future studies are needed to orientate better care in AF related to cancer.

## Author Contributions

LH and TM participated in writing the manuscript. SF conducted the writing and review of the manuscript. All authors contributed to the article and approved the submitted version.

## Conflict of Interest

The authors declare that the research was conducted in the absence of any commercial or financial relationships that could be construed as a potential conflict of interest.
